# Investigation of ABCA4 Missense Variants and Potential Small Molecule Rescue in Retinal Organoids

**DOI:** 10.1167/iovs.66.9.58

**Published:** 2025-07-22

**Authors:** Davide Piccolo, Paul Sladen, Rosellina Guarascio, Kalliopi Ziaka, Michael E. Cheetham

**Affiliations:** 1 UCL Institute of Ophthalmology, University College London, London, United Kingdom

**Keywords:** ABCA4, missense variants, retinal organoids, small compounds, misfolding

## Abstract

**Purpose:**

ABCA4-related retinopathy is the most common monogenic eye disorder in the world and is currently untreatable. Missense variants in *ABCA4* constitute ∼60% of causal ABCA4-related retinopathy variants, often resulting in misfolded or dysfunctional protein products. Despite their prevalence, the molecular mechanisms by which these missense mutations impair ABCA4 function are not fully understood, primarily due to limitations in suitable cellular models. In this study, we investigated the cellular and molecular consequences of ABCA4 missense variants using a human photoreceptor-like model system.

**Methods:**

We used CRISPR/Cas9 technology to introduce two ABCA4 missense misfolding variants, T983A and R2077W, which are associated with ABCA4-associated retinopathy, into control induced pluripotent stem cells (iPSCs). The iPSCs were differentiated into retinal organoids, characterized and treated with small molecules.

**Results:**

The expression level of ABCA4 missense proteins was reduced compared to WT ABCA4 suggesting the variants were degraded in a photoreceptor-like environment. The localization of the missense variants was also altered with negligible ABCA4 detectable in the retinal organoid outer segments compared to the isogenic control. Two small molecule compounds, AICAR and 4-PBA, previously identified as potential ABCA4 folding correctors in vitro, were tested for their ability to enhance ABCA4 traffic to the outer segment. The compounds did not appear to promote ABCA4 folding and traffic in photoreceptors and instead led to a decrease in *ABCA*4 transcript levels and protein.

**Conclusions:**

These data highlight that retinal organoids are an exquisite model to investigate pathogenic variants in ABCA4 and test small compounds for translation to the human retina.

The ATP-binding cassette, subfamily A, member 4 (ABCA4) protein is located in the outer segment disk rim^1^ and is essential for the clearance of toxic retinoid metabolites from the visual cycle.^2^ Without functional ABCA4 protein, the lipofuscin fluorophore A2E accumulates in the retinal pigment epithelium (RPE),^3^^,^^4^ causing RPE dysfunction and subsequent photoreceptor loss.^5^^–^^7^ Biallelic pathogenic variants in the *ABCA4* gene cause a range of phenotypically heterogeneous conditions defined as ABCA4-associated retinopathy, the most common inherited Mendelian eye disorder in the world.^7^^,^^8^ In its “classic” form, or Stargardt disease type 1 (STDG1), central vision loss manifests between adolescence and young adulthood; however, the age of onset and the severity of this condition vary, ranging from early-onset cone–rod dystrophy and retinitis pigmentosa–like to very late onset of mild disease similar to age-related macular degeneration.^7^

The *ABCA4* gene consists of 50 exons, on chromosomal locus 1p22.1.^8^ Over 2800 unique *ABCA4* variants have been identified since the discovery of the gene in 1997,^8^ with more than 2200 being pathogenic or likely pathogenic (https://databases.lovd.nl/shared/genes/ABCA4). The locus contains all classes of variants: missense, nonsense, insertion–deletion (indel), canonical and noncanonical splice site defects, deep intronic variants, and regulatory and structural variants.^7^ Over 60% are missense variants with single amino acid substitutions throughout the ABCA4 peptide.^9^ Because missense variants are associated with the majority of ABCA4-related retinopathy, their biochemical characterization using heterologous expression in cell culture has been of great interest, shedding light on their effect on folding, traffic, and/or function of the protein.^10^^–^^12^ However, in vitro systems do not fully replicate the complexity of photoreceptors, where endogenous ABCA4 is expressed, hence the need to study these variants in more context-specific models.

Several systems have been employed for the study of ABCA4-associated retinopathy. The first *Abca4* knockout animal model was reported in 1999.^3^ Although this model mimics a prominent pathological feature observed in humans, the accumulation of lipofuscin, photoreceptor degeneration, and delayed dark adaptation were not robustly replicated in the mouse model,^13^^–^^16^ probably because mice lack a macula. Two *Abca4* knock-in mouse models have also been generated to investigate specific protein defects. The *Abac4^L541P/A1038V^* knock-in complex allele is characterized by only a small trace of ABCA4 protein,^17^ and the *Abca4^N^^965S^* knock-in shows loss of substrate-dependent ATPase activity and increased localization to the inner segment (IS) of photoreceptors.^18^ Ectopic expression of ABCA4 missense variants c.5882G>A p.G1961E and c.2551G>A p.G851N in *Abca4* knockout mice has also been performed to further understand the cellular pathogenicity of missense variants. In particular, G1961E was predominantly detected in the outer segment (OS), whereas G851N was localized only in the IS. Transgenesis experiments in *Xenopus laevis* have also been used extensively for the description of missense variants in their ability to traffic toward the OS, in particular for the c.1804C>T p.R602W, c.1622T>C p.L541P, c.3113C>T p.A1038V, L541P/A1038V, and c.4468G>A p.C1490Y variants,^19^ emphasizing once again that misfolding and mistrafficking are common mechanisms underlying the pathogenicity of ABCA4 missense variants.

The use of retinal organoids to study ABCA4-associated retinopathy has been limited to study variants producing aberrant *ABCA4* mRNA.^20^^–^^22^ The most common severe disease-associated variant c.5461-10T>C, which leads to exon skipping and out-of-frame *ABCA4* transcripts, was corrected using antisense oligonucleotides to promote exon inclusion and restore wild-type (WT) RNA in retinal organoids.^23^ However, only recently have missense variants been studied in retinal organoids. The most common variant in *ABCA4* G1961E has been used as a paradigm variant for the optimization of base editing,^24^ and the complex genotypes c.[5461-10 T > C;5603 A > T, p. Asn1868Ile;4685 T > C, p.Ile1562Thr] and c.[5461-10 T > C, 5603 A > T, p.Asn1868Ile;5603 A > T, p.Asn1868Ile] were studied in retinal organoids.^25^

Here, we used clustered regularly interspaced short palindromic repeats (CRISPR)/Cas9 technology to produce and characterize induced pluripotent stem cell (iPSC)-derived retinal organoids carrying homozygous ABCA4 misfolding missense variants c.2947A>G p.T983A and c.6230C>T p.R2077W. We subsequently tested the potential of two small molecule compounds, 4-phenyl butyric acid (4-PBA) and 5-aminoimidazole-4-carboxamide ribonucleotide (AICAR), previously identified for their ability to enhance ABCA4 missense variants trafficking to the cell surface in vitro,^26^ to improve ABCA4 protein traffic in retinal organoids and evaluate their feasibility as a missense-variant–independent therapeutic approach for the treatment of ABCA4-associated retinopathy.

## Methods

### Homology-Directed Repair With CRISPR/Cas9

CRISPR/Cas9 gene editing was accomplished by following and combining previous protocols.^27^^–^^31^ CRISPR/Cas9 guides were designed to direct the Cas9 endonuclease to cut as close as possible to the region of interest. The design of the guide RNAs (gRNAs) was completed on (https://www.benchling.com/), a web-based software enabling the design of optimal gRNAs (Supplementary Table S1). The gRNAs were selected for minimal off-target and maximum on-target activity, according to the algorithms developed by Hsu et al.^32^ and Doench et al.^33^ Off-target analysis was performed using Off-Spotter (Thomas Jefferson University, Philadelphia, PA, USA), and no major unwanted off-targets were identified. All of the off-targets found were either in pre-mRNA or intronic regions of non–inherited retinal disease genes, with a number of mismatches ≥ 2 (Supplementary Fig. S1). Single-stranded DNA (ssDNA) templates were designed to asymmetrically overlap the double-stranded DNA (dsDNA) break site induced by gRNA and Cas9 and to introduce the c.2947A>G (T983A) and the c.6229C>T (R2077W) changes, while at the same time introducing synonymous changes at the Sp.Cas9 gRNA protospacer-adjacent motif (PAM) site, to prevent further cleavage of the target region (Supplementary Table S2).

### Nucleofection

Following the design of the CRISPR/Cas9 strategy, previously published control (WT) iPSCs^34^ were nucleofected with ribonucleotide protein (RNP) complexes containing the *ABCA4* targeting gRNA and the specific ssDNA templates. On the day of editing, the iPSC media were changed to Gibco StemFlex (Thermo Fisher Scientific, Waltham, MA, USA), and 4 µM Rho-associated protein kinase inhibitor (ROCKi) was added at least 2 hours prior to dissociation. All of the transfection reagents were prepared during this time according to the manufacturer's instructions. CRISPR RNA (crRNA), trans-activating CRISPR RNA (tracrRNA), and ssDNA donors were resuspended in nuclease-free double-distilled water (ddH_2_O) to 100 µM. tracrRNA and crRNA were then combined to form a 50 µM gRNA complex solution and heated to 95°C for 5 minutes. Then, 3 µL of gRNA complexes was combined with 2 µL of 61 µM Alt-R Cas9 enzyme (Integrated DNA Technologies, Coralville, IA, USA) to generate RNP complexes, followed by incubation at room temperature for 20 minutes. Cells were then counted and centrifuged at 200*g* for 5 minutes. Following centrifugation, the supernatants were carefully removed, and the cells were resuspended in P3 Primary Cell 4D-Nucleofector solution (Lonza, Basel, Switzerland) before adding 5 µL of RNP solution, 2 µL of 100 µM ssDNA, and 4 µL Electroporation Enhancer (Integrated DNA Technologies). The iPSC–RNP mix was then nucleofected using Lonza 4D-Nucleofector with X-unit and the program CA-137. Following nucleofection, cultures were maintained until iPSC colonies emerged. Individual clones were mechanically isolated and placed into individual wells of a Geltrex-coated 12-well plate (Thermo Fisher Scientific). Clonal iPSC lines were subsequently expanded, and the CRISPR/Cas9 target region was expanded and analyzed by Sanger sequencing using *ABCA4* primers (Supplementary Table S3) to detect the presence or absence of induced mutations.

### Genomic DNA Extraction

Total genomic DNA (gDNA) was extracted using the Wizard gDNA Purification Kit (Promega, Madison, WI, USA) following the manufacturer's guidelines. Cells were washed with Dulbecco’s Phosphate Buffered Saline (DPBS) and lysed using 250 µL of Wizard SV Lysis Buffer (Promega). Individual Wizard Minicolumns (Promega) were used to collect samples, and total gDNA was quantified using spectrophotometry with a NanoDrop 2000 (Thermo Fisher Scientific).

### Differentiation of iPSC Into Three-Dimensional Retinal Organoids

The retinal organoid differentiation protocol used here was an adaptation from previously published protocols for three-dimensional retinal organoids.^35^^–^^37^ iPSCs were maintained until 90% to 95% confluency, and then medium without fibroblast growth factor (FGF; Thermo Fisher Scientific) was added to the culture for 2 days (day 1 [D1] and D2 of differentiation). A neural induction period was then begun using pro–neural induction media. A pulse feeding with bone morphogenetic protein 4 (BMP4) was started at day 6 and terminated at day 16. From day 20 it was possible to appreciate the formation of lightly pigmented RPE cells that contained neuroretinal vesicles (NRVs), characterized by retinal neuroepithelium. When the NRVs had formed, checkerboard scraping was performed, usually at day 26.^38^^,^^39^ This consists of breaking the iPSC-derived tissue sheets with a sterile blade into smaller pieces to generate numerous retinal aggregates. These structures were then kept in suspension in large Petri dishes, and different media were used over time: retinal differentiation medium (RDM) from days 26 to 33; retinal maturation media 1 (RMM1) from days 34 to 70; RMM2 from days 71 to 100; and RMM3 from day 100 to the end of the differentiation. Then, 1 µM retinoic acid was added at day 50 through day 70, reduced to 0.5 µM at day 70 until day 100, and removed after this time point.

### RNA Extraction From Organoids

RNA was extracted from single snap-frozen organoids using the RNeasy Mini Kit (QIAGEN, Hilden, Germany). Frozen organoids were mechanically homogenized in Buffer RLT (QIAGEN) supplemented with 143-mM β-mercaptoethanol. The lysates where then moved to an RNeasy spin column (QIAGEN). Contamination with gDNA was removed by an on-column DNase digestion with the RNAse-Free DNase Set (QIAGEN). Total RNA was quantified with the NanoDrop 2000.

### Complementary DNA Synthesis

First-strand complementary DNA (cDNA) was produced from RNA using a Tetro cDNA Synthesis Kit (Bioline, Memphis, TN, USA). A reagent master mix was prepared as per the manufacturer's instructions, and 100 ng of RNA was added. The samples were then incubated at 45°C for 1 hour and at 85°C for 5 minutes.

### Polymerase Chain Reaction

Primers for DNA analysis were designed manually and validated using the BLAT Search Genome (https://genome.ucsc.edu/cgi-bin/hgBlat) for correct annealing and Oligo-calculator (http://biotools.nubic.northwestern.edu/OligoCalc.html) in order to check self-complementarity . A master mix was prepared using Q5 High-Fidelity 2X Polymerase (New England Biolabs, Ipswich, MA, USA), combined with 0.5 µM forward and reverse primers, and 100 ng of DNA was used as a template. PCR amplification was then confirmed on a 1% to 2% (w/v) agarose (Bioline) gel containing 0.005 % (v/v) SafeView nucleic acid stain (Applied Biological Materials, Vancouver, Canada) and was subsequently visualized using ChemiDoc XRS+ with ImageLab software (Bio-Rad, Hercules, CA, USA). The QIAquick Gel Extraction Kit (QIAGEN) was used to extract DNA from agarose gels following the manufacturer's instructions.

### Sequencing

DNA samples were sequenced using Sanger sequencing by Source BioScience (Nottingham, UK).

### Reverse Transcription Quantitative Polymerase Chain Reaction

Reverse transcription quantitative polymerase chain reaction (RT-qPCR) using SYBR Green was used to determine levels of gene expression. cDNA (4 ng) was mixed with 10 µL of 2X LabTAQ Green Mix Hi Rox (Labtech, Rotherham, UK) and 1 µL of 10 µM forward and reverse primers to a total volume of 25 µL with ddH_2_O. Each sample was prepared in triplicate. Reactions were then loaded onto a MicroAmp Optical 96-well reaction plate (Thermo Fisher Scientific); the plate was centrifuged, and the reaction was performed using a QuantStudio 6 Flex Real-Time PCR system (Thermo Fisher Scientific). Raw data were then exported into Excel (Microsoft, Redmond, WA, USA). Target genes were normalized to the geometric mean of two reference genes (*ACTIN* and *GAPDH*).

### Primer Design and Amplicon Validation

Primer pairs designed for relative quantification can be found in Supplementary Table S4. Primers were obtained from three different sources: manually designed; obtained from PrimerBank (https://pga.mgh.harvard.edu/primerbank/); or primers already used and validated within the Cheetham lab. Primers were then validated using the BLAT Search Genome (https://genome.ucsc.edu/cgi-bin/hgBlat) for correct annealing and Oligo Calculator (http://biotools.nubic.northwestern.edu/OligoCalc.html) in order to check self-complementarity.

### Fixation, Embedding, and Cryosectioning of Retinal Organoids

Organoids were washed once with PBS and collected into Bijou vials. Samples were then fixed with 2% paraformaldehyde and 5% sucrose (Sigma-Aldrich, St. Louis, MO, USA) in PBS for 15 minutes at 4°C. Following fixation, organoids were infiltrated with sucrose as follows: 7.5% sucrose in PBS for 30 minutes at 4°C; 15% sucrose in PBS for 30 minutes at 4°C; and 30% sucrose in PBS for at least 1 to 2 hours or until the organoids reached the bottom of the vial. Finally, the solution was removed, and organoids were incubated in 7.5% porcine gelatin (Sigma-Aldrich) and 10% sucrose in PBS for 15 minutes at 37°C. Samples were then transferred to a cryomold, frozen in dry ice, and stored at −80°C until the day of processing. Organoids were cryosectioned at 10 µm using a Leica cryostat (Leica Biosystems, Nussloch, Germany) and stored at −20° until further processing.

### Immunofluorescence on Retinal Organoids

Gelatin-embedded sections were washed with 1× PBS and incubated in blocking solution containing 10% (v/v) fetal bovine serum and 0.1% Triton X-100 (v/v) (Sigma-Aldrich) in PBS for 1 hour at room temperature, thus preventing non-specific binding. Following blocking, sections were incubated with the primary antibodies: ABCA4 (ab77285, 1:1000; Abcam, Cambridge, UK); ABCA4 (MAB2240, 1:1000; Merck, Rahway, NJ, USA); Rho1D4 (University of British Columbia [UBC], BC, Canada) (1:1000); anti-arrestin-C (MABN2636, 1:250; Sigma-Aldrich); and Tom20 (Sc-17764 1:150; Santa Cruz Biotechnology, Dallas, TX, USA). These were mixed with blocking solutions diluted 1:2 in PBS for 1 hour at room temperature or overnight at 4°C. The blocking solution was washed away two times with PBS and incubated for 1 hour with the appropriate fluorescently conjugated secondary antibodies diluted in blocking/permeabilizing solution at room temperature in the dark and/or with Invitrogen Wheat Germ Agglutinin (WGA; W32464, 1:1000; Thermo Fisher Scientific) and Invitrogen Phalloidin 488 (1:1000; Thermo Fisher Scientific). After three washes, the samples were incubated for 5 minutes with 5 µg 4′,6-diamidino-2-phenylindole (DAPI; Sigma-Aldrich) in PBS for nuclear staining. Cells were rinsed with PBS, and the chambers and the glue were removed. Slides were mounted with glass coverslips using Dako Fluorescence Mounting Medium (Agilent, Santa Clara, CA, USA) and were stored at 4°C.

### Organoid Protein Extraction Using Radioimmunoprecipitation Assay Buffer

Organoids samples were gently washed in cold PBS and lysed in radioimmunoprecipitation assay (RIPA) buffer supplemented with 2% (v/v) protease inhibitor cocktail (Sigma-Aldrich) and 1% phosphatase inhibitor cocktail (Sigma-Aldrich) for at least 30 minutes at 4°C; they were vortexed every 10 minutes. To facilitate tissue homogenization, samples were mechanically destroyed and pounded with a micropestle.

### Western Blot

Following protein extraction and quantification, Laemmli sample buffer (5×) containing 250-mM Tris–HCl (pH 6.8, 50%, v/v), glycerol (10%, w/v), sodium dodecyl sulfate (SDS), 525-mM dithiothreitol (DTT), and bromophenol blue was added to each lysate, and 20 µg of proteins was resolved via SDS–polyacrylamide gel electrophoresis (PAGE) on 8% acrylamide gels. A PageRuler Plus Prestained Protein Ladder (Thermo Fisher Scientific) was also loaded as a molecular weight marker. Loaded protein samples were run at 80 V until they were placed in the stacking gel and then the voltage was increased to 100 to 120 V when they reached the resolving gel. Proteins were then electrophoretically transferred onto a 0.45-µm polyvinylidene difluoride (PVDF) membrane via wet transfer using transfer buffer, run for 90 minutes at constant 100 V. Membranes were blocked in 5% (w/v) non-fat dried milk in PBS–Tween (PBST, 0.1%, w/v; Tween-20 in PBS) for 1 hour at room temperature to prevent non-specific binding. Membranes were then incubated overnight with primary antibody diluted in 5% (w/v) milk–PBST at 4°C in agitation: ABCA4 (ab72955, 1:1000; Abcam), GAPDH (60004-1-lg, 1:10,000; Proteintech, Rosemont, IL, USA), vinculin (4650S, 1:1000; Cell Signaling Technology, Danvers, MA, USA), and rhodopsin (129910, 1:1500; Genetex, Irvine, CA, USA). The following day the membranes were washed with PBST three times for 10 minutes each and then incubated for 1 hour with the secondary antibody coupled to horseradish peroxidase in 5% (w/v) milk–PBST. After incubation, the membranes were washed three times for 5 minutes each and developed with Clarity or Clarity Max ECL Western Blotting Substrates (Bio-Rad). We used two different reference proteins for the western blot: GAPDH and vinculin were used as a general protein markers, and, to make the analysis more stringent, we used Rho, which is specific for photoreceptors and the OS. The chemiluminescence detection was performed using the ChemiDoc MP Imaging System (Bio-Rad), and the signal intensity was evaluated using ImageJ (National Institutes of Health, Bethesda, MD, USA).

## Results

### Generation of Homozygous T983A and R2077W ABCA4 iPSC Via CRISPR/Cas9 Technology

**Figure 1.**
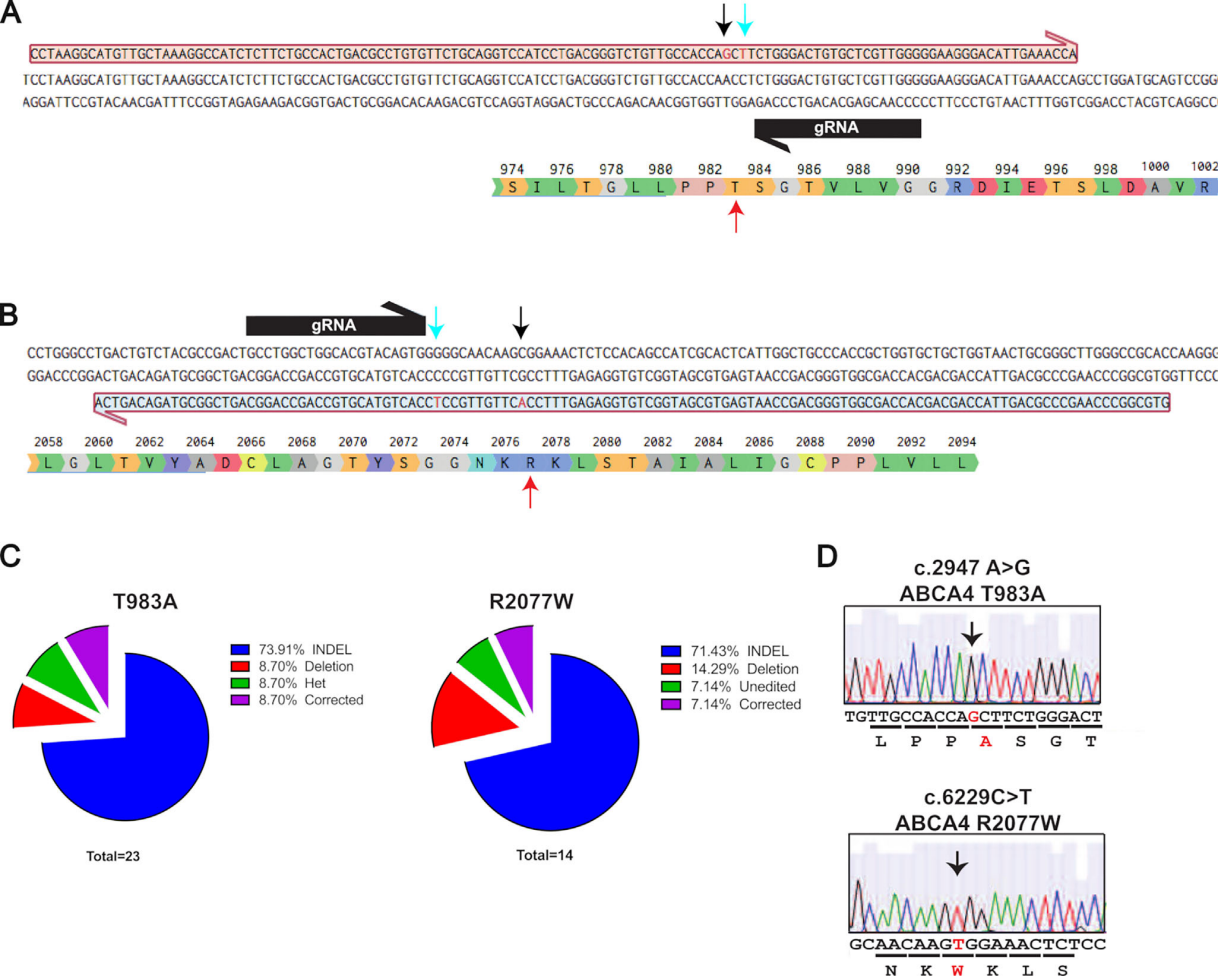
CRISPR/Cas9-directed gene editing of *ABCA4*. (**A**) The *red box* shows the ssDNA, aligned with *ABCA4* genomic sequence, carrying c.2947A>G T983A (*black arrow*, *red letter*) and the change in the PAM sequence (*cyan arrow*, *red letter*). The gRNA is shown as a *black box*, and the *red arrow* points toward the targeted amino acid in position 983. (**B**) The *red box* shows the ssDNA, aligned with *ABCA4* genomic sequence, carrying the c.6229C>T R2077W (*black arrow*, *red letter*) and the change in the PAM sequence (*cyan arrow*, *red letter*). The gRNA is shown as a *black box*, and the *red arrow* points toward the targeted amino acid in position 2077. (**C**) Pie charts depict the distribution of various editing events that occurred after CRISPR/Cas9, selection, and sequencing of the clones. (**D**) Sanger sequencing results for the iPSC single colonies show the right single nucleotide changes in homozygosity.

CRISPR/Cas9 gene editing with a ssDNA repair template was used to generate knock-in cell lines carrying either homozygous c.2947 A>G (T983A) or c.6229 C>T (R2077W) ABCA4 variants (Figs. 1A, 1B). These variants were previously characterized and shown to be endoplasmic reticulum (ER) retained with reduced trafficking to the plasma membrane in HEK293 cells.^26^ No off-targets were found with 0 mismatch for the gRNAs when analyzed using Off-Spotter (Supplementary Fig. S1). Two clones out of a total of 23 screened for T983A were successfully edited, and only one clone out of 14 clones for R2077W was successfully edited. The majority of the remaining clones contained indels (around 70% of the events), but we also detected some clones with deletions or heterozygous knock-in for the base changes or that were unedited (Fig. 1C). Clones that were correctly edited were identified by Sanger sequencing having G homozygosity at position c.2947 (T983A), or T homozygosity at position c.6229 (R2077W) and the introduction of a synonymous change at the Cas9 PAM site to prevent further DNA cleavage (Fig. 1D).

### Retinal Organoid Differentiation

**Figure 2.**
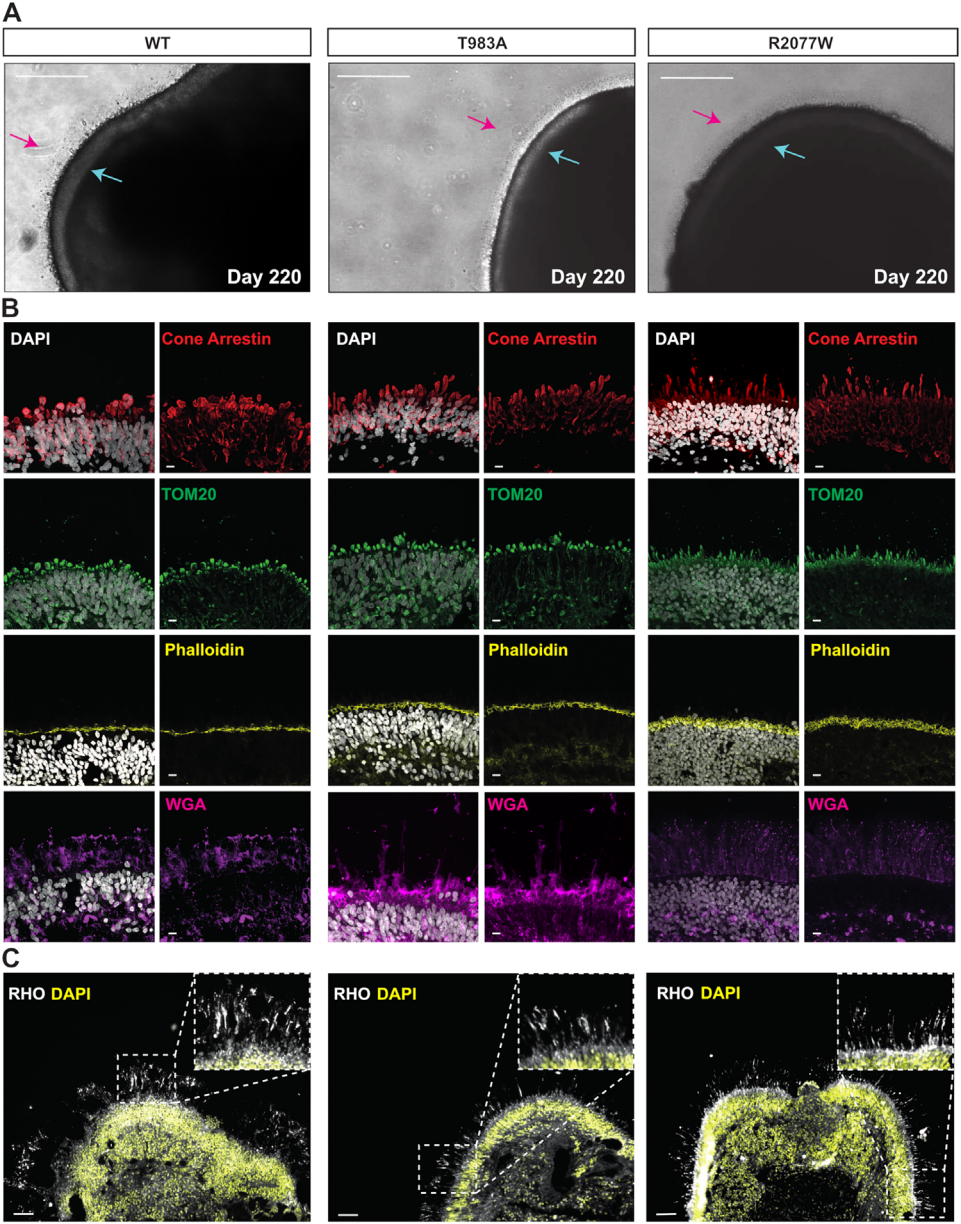
Characterization of ABCA4 edited retinal organoids at D220. (**A**) At D220, the three different retinal organoids lines (WT isogenic control, T983A, and R2077W) reached full maturity. *Scale bars*: 250 µm. (**B**) Immunofluorescence analysis of WT, T983, and R2077W lines expressing the mature cone marker cone arrestin. Mitochondria staining (TOM20) was used to visualize the IS, phalloidin was used as the outer limiting membrane marker, and WGA was used to identify the OS. DAPI staining is shown in *gray*. *Scale bars*: 10 µm. (**C**) The overall retinal organoid structure is shown at lower magnification. The laminated neuroretina-like outer structure was visible by DAPI staining (*yellow*), highlighting the ONL of photoreceptors; Rho-positive hair-like structures (*gray*) can be seen protruding from the laminated area (*insets*), indicating a well-preserved OS. *Scale bars*: 50 µm.

Isogenic WT, T983A, and R2077W iPSC lines were differentiated into retinal organoids using a routinely employed protocol in our research group (Supplementary Fig. S2A).^40^ The retinal organoids were grown for 220 days (D220), and their development was monitored by brightfield imaging. As early as D70, a phase-bright neuroepithelium was observed across organoids from all iPSC lines. From D120 onward, all retinal organoids displayed evidence of OS formation, protruding from the apical edge (Supplementary Fig. S2B). By the final day of differentiation (D220), all organoids exhibited a typical mature morphology, with a clear phase-bright outer nuclear layer (ONL) and a thick brush-border layer of OS (Fig. 2A). Immunohistochemistry confirmed that all of the retinal organoids showed expression of key photoreceptor markers, including cone arrestin and Rho. No major differences were detected in the variant organoid morphology compared to WT when staining for actin filaments (phalloidin) or mitochondria (TOM20), that highlighted the outer limiting membrane and the IS, respectively. The photoreceptor OS was also well formed in the variant lines, as defined by Rho immunoreactivity and wheat germ agglutinin (WGA), which stains the interphotoreceptor extracellular matrix. Overall, the immunofluorescence staining confirmed successful differentiation, correct lamination, and development of the T983A and R2077W lines into retinal organoids morphologically resembling the controls (WT) (Figs. 2B, 2C).

### ABCA4 T983A and R2077W Mutant Retinal Organoids Exhibit Reduced Levels of ABCA4 Protein

**Figure 3.**
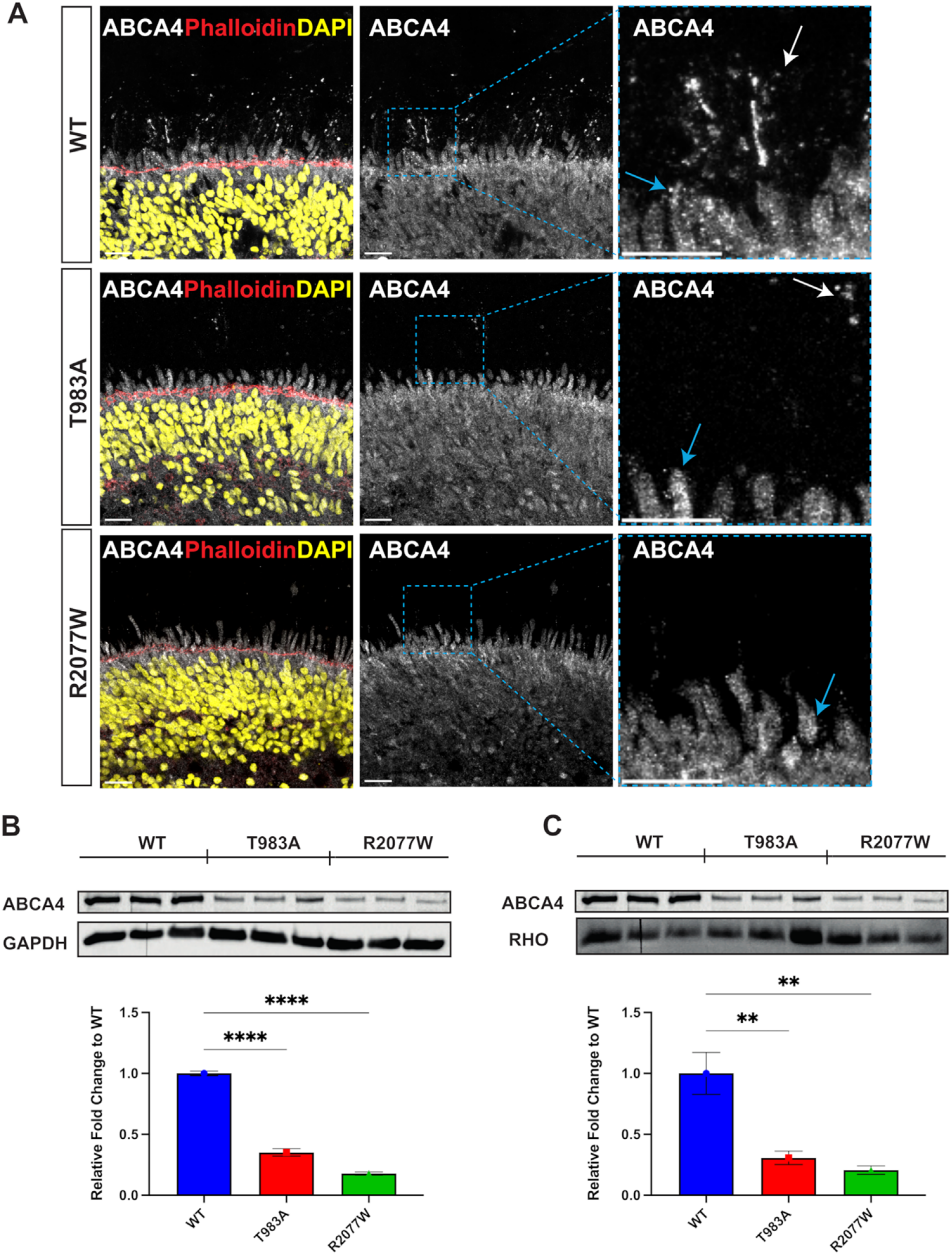
Characterization of ABCA4 in T983A and R2077W retinal organoids. (**A**) Immunofluorescence analysis of ABCA4 WT, T983A, and R2077W retinal organoids using the N-terminus 5B4 antibody. WT ABCA4 localized in the OS of photoreceptors (*white arrow*) and IS (*cyan arrow*). T983A ABCA4 immunoreactivity was reduced, and only a few ABCA4-positive spots could be seen in the OS (*white arrow*) and IS (*cyan arrow*). R2077W ABCA4 immunofluorescence signal was reduced, and no OS-positive signal was observed, but only in the IS (*cyan arrow*). *White scale bar*: 20 µm. (**B**, **C**) Western blot and quantification from 20-µg protein lysate from two retinal organoids pooled together per well. GAPDH was used as a general reference protein (**B**), and Rho was used as photoreceptor and OS differentiation reference (**C**). *Error bars* are SEM. One-way ANOVA followed by Dunnett's post hoc test was performed only against WT ABCA4 samples. **P < 0.01, ****P < 0.0001 (*n* = 3 from different differentiations).

ABCA4 localization in homozygous T983A and R2077W retinal organoids was analyzed at D220, as *ABCA4* expression has been shown to steeply increase until D120 with a gradual peak after D200,^23^ coinciding with photoreceptor maturation. Confocal microscopy analyses using ABCA4 5B4 N-terminus antibody confirmed that WT ABCA4 localized mainly in the OS of photoreceptors but also in the IS. In contrast, ABCA4 immunoreactivity in T983A and R2077W retinal organoids was greatly reduced; only a few ABCA4 positive spots were detected in the OS and IS of the T983A samples, and even fewer were detected in the OS and IS of R2077W (Fig. 3A). Similar results were obtained using ABCA4 3F4 C-terminus antibody, and the presence of retinal organoid OS structures was highlighted by differential interreference contrast microscopy of the samples (Supplementary Fig. S3). *ABCA4* transcript levels were analyzed by RT-qPCR. No significant difference was observed in *ABCA4* transcript levels between the WT and variant organoids (Supplementary Fig. S4). We then analyzed the steady-state protein levels by western blot. The levels of ABCA4 from the variant organoids were significantly decreased compared to the WT. The T983A variant protein expression was reduced by 70% compared to the WT and the R2077W by 90%, suggesting that these ABCA4 variant proteins are recognized by the cell protein degradation machinery and eliminated (Figs. 3B, 3C). Original uncropped blots are shown in Supplementary Figure S5. Collectively, these data support a posttranscriptional decrease in ABCA4, probably caused by protein degradation and failed traffic of ABCA4 to the OS for these variants, and they suggest that these organoids can be used to test protein folding correctors.

**Figure 4.**
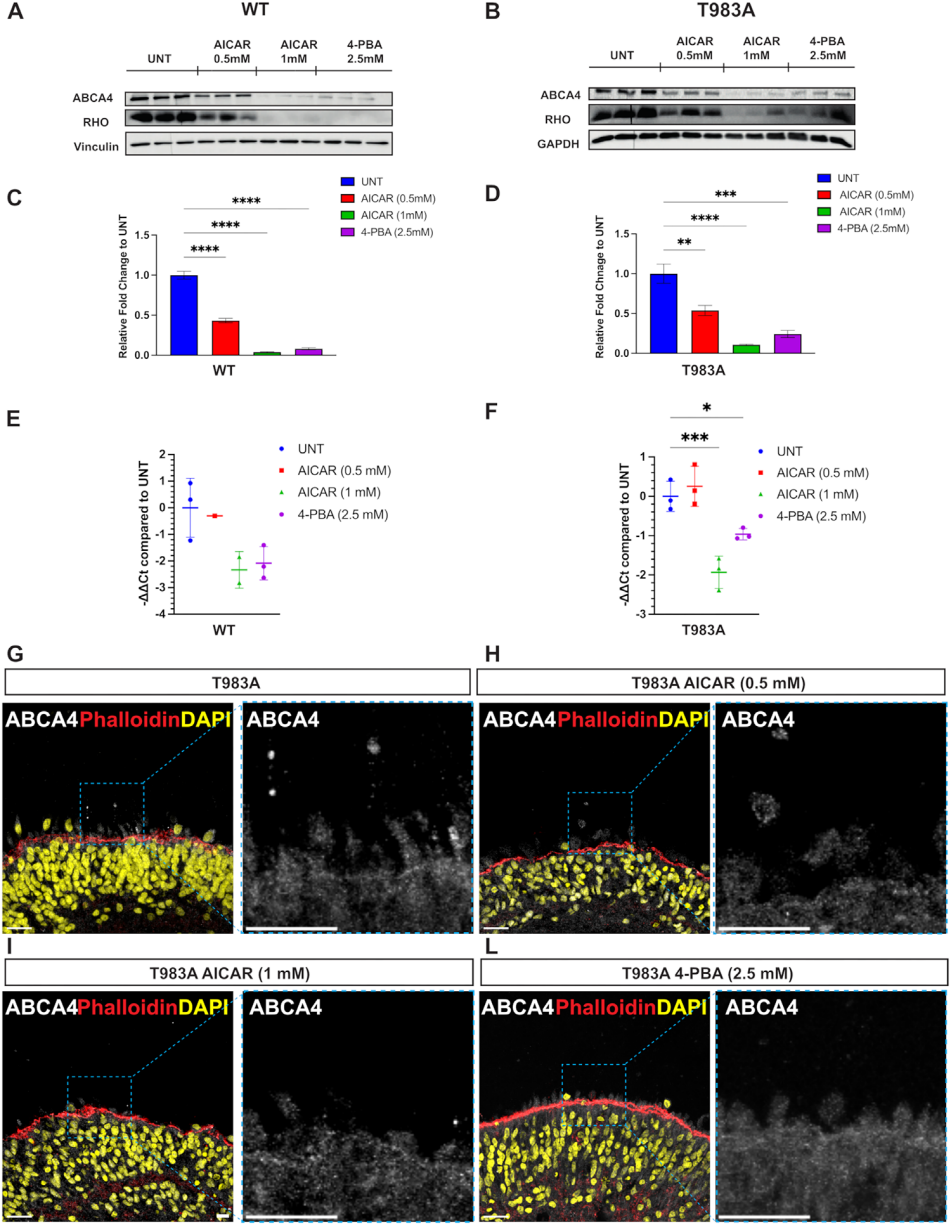
AICAR and 4-PBA effects on ABCA4 in retinal organoids. (**A**–**D**) Western blot and quantification of ABCA4 WT (*left*) and T983A (*right*) from 20-µg protein lysate of two retinal organoids pulled together per well. GAPDH, vinculin, and Rho were used as reference protein controls. Quantification of the western blot is also shown. Both ABCA4 WT and T983A showed a significant decrease in protein levels following AICAR and 4-PBA treatments. In particular, 0.5-mM AICAR halved ABCA4 protein levels, and 1-mM AICAR and 2.5-mM 4-PBA reduced protein levels. *Error bars* are SEM. One-way ANOVA followed by Dunnett's post hoc test was performed only against the untreated ABCA4 sample (*n* = 3 from different differentiations). (**E**, **F**) WT and T983A *ABCA4* transcript levels following AICAR (0.5-mM and 1-mM) and 4-PBA (2.5-mM) treatment. *ABCA4* expression was first normalized to the geometric mean of two reference genes, *GAPDH* and *ACTIN*, before normalization to the untreated (UNT) condition. *Error bars* are SD. One-way ANOVA followed by Dunnett's post hoc test was performed only against the untreated ABCA4 sample. (**G**–**L**) Immunofluorescence analysis of ABCA4 T983A retinal organoids (**G**). Only a small fraction of ABCA4 reached the OS (**H**–**L**). No major effect was observed on ABCA4 protein localization. *White scale bar*: 20 µm. *P < 0.05, **P < 0.01, ***P < 0.001, ****P < 0.0001.

### AICAR and 4-PBA Negative Effect on ABCA4 in Retinal Organoids

We previously reported that the small molecules 4-PBA and AICAR can enhance the traffic of missense misfolded ABCA4 variants, including T983A and R2077C, toward the plasma membrane in vitro.^26^ Therefore, we tested the effect of these molecules in retinal organoids. WT and T983A retinal organoids were treated for a total period of 2 weeks with AICAR (0.5-mM and 1-mM) and 4-PBA (2.5-mM), starting at D220 and finishing at D234. Samples were then collected for western blot, RT-qPCR analysis, and immunofluorescence. There were no clear differences in the phase contrast morphology of treated and untreated retinal organoids (Supplementary Fig. S6). The level of ABCA4 protein in treated WT and T983A retinal organoids was investigated, and a significant reduction in WT ABCA4 and T983A steady-state protein levels was observed following treatment (Figs. 4A, 4B). The reduction following the AICAR treatment appeared to be dose dependent, with 60% of WT and 50% of T983A protein levels reduced for 0.5-mM AICAR compared to untreated, but reaching more than 90% reduction for 1-mM AICAR in both genotypes. 4-PBA also had a strong effect, reducing the ABCA4 protein levels by 90% and 80% in WT and T983A, respectively (Figs. 4C, 4D). Original uncropped blots can be found in Supplementary Figure S7. Interestingly, following AICAR and 4-PBA treatment, Rho protein levels were also reduced (Figs. 4A, 4B), but the other reference protein controls, vinculin and GAPDH, were unaltered following AICAR and 4-PBA treatments in both WT and T983A lines, suggesting this might be a photoreceptor protein-specific effect. To better understand the effect of these compounds on retinal organoids, *ABCA4* transcript levels were analyzed by RT-qPCR following AICAR and 4-PBA treatments in WT and T983A retinal organoids. No effect on transcript levels were observed following 0.5-mM AICAR treatment compared to the WT line. In contrast, a strong reduction in *ABCA4* transcript levels was observed following 1-mM AICAR treatment in WT organoids compared to the untreated, and a similar effect was also obtained with 4-PBA (Fig. 4E). Nevertheless, similar results were obtained from T983A retinal organoids, where no differences were observed following treatment with 0.5-mM AICAR compared to untreated, whereas a significant strong reduction in *ABCA4* transcript was observed with 1-mM AICAR and 2.5-mM 4-PBA, suggesting that these concentration of drugs have an effect on *ABCA4* at the transcriptional level (Fig. 4F). The effect of the drugs on T983A ABCA4 localization and expression was investigated by immunofluorescence. In the untreated T983A retinal organoids, ABCA4 localization was characterized by few ABCA4 immunoreactive areas detected in the OS and IS. Following treatments with AICAR or 4-PBA, T983A ABCA4 immunoreactivity was absent, and no positive signal was observed, in agreement with the western blot data (Figs. 4G–L). The retinal organoids were also stained for Rho to make sure that the OS were preserved during processing. Rho staining was observed in all the conditions, but the staining intensity appeared to be reduced compared to untreated organoids, in agreement with western blot data (Supplementary Fig. S8). Given the changes in photoreceptor protein levels, we examined if there were changes in photoreceptor number. No significant change in ONL thickness was detected in treated organoids compared to untreated (Supplementary Fig. S9), suggesting that there was no photoreceptor cell death over the time course of the experiment.

## Discussion

The investigation of pathogenic ABCA4 missense variants has been mainly carried out using heterologous expression in various cultured cell lines, such as HEKs, CHOs, and COS7.^11^^,^^12^^,^^26^^,^^41^ Although these cells have similar endoplasmic reticula and traffic to the plasma membrane, they do not fully recapitulate the correct gene dosage and potential specializations of the photoreceptor protein folding or quality control machinery, as well as specialized compartments such as the outer segment. Knock-in mouse lines have also been generated, including c.2894A>G, p.N965S,^18^ and the double mutant [L541P;A1038V].^17^ Although these models accumulate lipofuscin, unlike the human disease they show no retinal degeneration up to 12 months of age. The human retina is enriched in cones, whereas the mouse retina is characterized by a low cone percentage (<3%), and this could compromise modeling human maculopathies.^42^ Moreover, the large number of variants and their uncertain impact on the severity of the disease have made in vivo models cost-ineffective systems for the study of disease-associated missense variants.

Retinal organoids derived from iPSCs have emerged as a powerful model system for investigating inherited retinal diseases. To date, they have mainly been used to study variants producing aberrant *ABCA4* mRNA and to identify potential targets for splicing–modulating therapies such as antisense oligonucleotides.^20^^,^^21^^,^^23^ More recently the most common variant in *ABCA4* G1961E has been used as a paradigm variant for the optimization of base editing in retinal organoids.^24^ The use of retinal organoids has also enabled the interrogation of more complex genotypes, such as c.[5461-10 T > C;5603 A > T, p. Asn1868Ile;4685 T > C, p.Ile1562Thr] and c.[5461-10 T > C 5603 A > T, p.Asn1868Ile;5603 A > T, p.Asn1868Ile], providing insights into genotype–phenotype correlations.^25^

Here, healthy control iPSCs were gene edited using CRISPR/Cas9, producing two different ABCA4 variant lines homozygous for the T983A and R2077W variants. Given the fact that the majority of ABCA4 patients are heterozygous or compound heterozygous, the advantage of gene editing becomes clear in this context, as it enables the isolated study of a variant in homozygosity without the presence of another variant allele being present. ABCA4 missense variants T983A and R2077W showed reduced ABCA4 protein steady-state levels in retinal organoids compared to WT organoids, with the R2077W variant being lowest. This reduction diverged with the overexpression cellular model, where T983A and R2077W variants were present at levels similar to those of WT ABCA4 as determined by solubilization in SDS,^11^^,^^26^ suggesting that they were subject to specific degradation mechanisms that do not occur in model cell lines but which occur in photoreceptors at endogenous levels of expression as opposed to overexpression in cells. No significant differences in transcript levels were observed among the lines, confirming that those variants affect *ABCA4* gene at the post-transcriptional level.

Recent studies have shown that some missense variants can directly impact pre-mRNA splicing.^43^ To investigate this, we used SpliceAI to predict the effects of variants T983A and R2077W on splicing and confirmed that these variants are not predicted to have any effect on splicing. Confocal microscopy analysis clearly highlighted how WT ABCA4 localizes in the OS of photoreceptors, but also in the biosynthetic IS, in agreement with published data from mouse retina showing that ABCA4 predominantly localizes to the OS with a fraction also detectable in the IS.^18^ By contrast, T983A and R2077W ABCA4 immunoreactivity was reduced, with only few spots detectable in the OS of the T983A model and no signal in the OS of the R2077W model, despite these organoids having Rho-positive OS, suggesting a failure of the proteins to traffic to this specific subcompartment of the photoreceptors, most likely due to ER retention in the IS and protein degradation. Indeed, when these variants were expressed in HEK and CHO cell lines they were distributed mainly in a reticular pattern indicative of misfolded protein sequestered in the ER and showed reduced traffic toward the plasma membrane.^9^^,^^26^

Another variant, N965S, is distributed between vesicular structures, typical of WT ABCA4 protein, and has an ER pattern when expressed in COS7 cells. In a homozygous knock-in mouse model of this variant, it exits the ER (65% of the total) and traffics to the photoreceptor outer segments similar to in vitro studies.^18^ The variant G1961E is also of interest, as it results in mild disease in patients, but in vitro studies have attributed a strong deleterious effect for the variant protein when analyzed for substrate binding and ATPase activity.^11^^,^^41^ However, this variant seems to reach the OS when ectopically expressed in *ABCA4^–/–^* mouse retinae.^44^.

Protein sorting and trafficking in photoreceptor cells represent an important and interesting paradigm. Photoreceptor cells are highly polarized neurons undergoing constant renewal of their OS membranes. OS resident proteins are first synthesized in the IS and then transported to the OS through the connecting cilium; however, our understanding of the protein-specific traffic mechanism is somewhat limited.^45^ Rho is the most abundant protein of rod OS and follows a conventional path from the ER, where it is synthesized, to the Golgi complex and trans-Golgi. From the Golgi, Rho is then transported to the base of the cilium in secretory vesicles that are referred to as Rho transport carriers and from there to the OS.^46^ In mouse rods, it has been suggested that Rho traffics to the plasma membrane before transport to the cilia membrane.^47^ A specific targeting signal has been identified at the C-terminus of Rho, which includes the last four amino acids with the VXPX motif.^48^ Mutations or deletions of these amino acids leads to mislocalization of Rho from the OS and leads to severe forms of retinal degeneration.^49^ The only OS-specific protein containing this motif apart from Rho is retinol dehydrogenase (RDH8), and the motif is required for correct OS delivery of this enzyme.^50^

Another protein that contains a targeting signal different from the VXPX motif is peripherin/RDS, where a 10-amino-acid sequence has been identified with OS targeting capability, and a specific valine at position 332 was shown to be critical for peripherin targeting.^45^ Moreover, peripherin/RDS has been shown to be transported to the plasma membrane using an unconventional secretory pathway, potentially bypassing the Golgi apparatus.^51^ In contrast to these proteins, little is known about other transmembrane proteins residing within the OS. In the case of ABCA4, it has only been shown that this protein is Endo H sensitive and therefore follows an unconventional secretory pathway to reach its destination directly from the ER in mice.^52^ However, nothing is known about ABCA4 transport from the IS to OS. Cell-based systems coupled with retinal organoids also hold tremendous potential to study ABCA4 trafficking to the connecting cilium and to the OS.

Gene editing of iPSCs and human retinal organoids have been used to study the rescue of aberrant *ABCA4* mRNA splicing using antisense oligonucleotides.^20^^,^^23^ Here, we studied the effect of small compounds on ABCA4 missense variants in retinal organoids. We tested previously identified potential folding correctors, AICAR and 4-PBA, for their ability to improve ABCA4 folding and traffic.^26^ We treated the organoids for 2 weeks based on an assessment that improved folding and trafficking to the OS might take time to increase to detectable levels, compared to the 48-hour treatment in the cell model.^26^ Both compounds decreased WT and T983A protein levels in the organoids at all of the concentrations tested. Rho levels were also reduced but not GAPDH and vinculin, suggesting a potentially specific effect on photoreceptors. The reasons for the AICAR and 4-PBA effect on ABCA4 and Rho are not clear but do not appear to be mediated by acute photoreceptor cell death, as we did not observe any changes in outer nuclear layer thickness.

The lower concentrations of AICAR did not have any effect on *ABCA4* transcript levels, whereas there was a reduction of more than 50% on the WT and T983A ABCA4 proteins, suggesting an effect on the protein or translation downstream of transcription. AICAR slows down protein translation^53^; thus, it is possible that the treatment has a general slowing effect in protein translation affecting predominantly photoreceptor proteins, potentially because they are the first cell layers in contact with the compound or they are extremely biosynthetic cells.

4-PBA also reduced *ABCA4* transcript levels in T983A retinal organoids, suggesting that the effect on the protein level could be a downstream effect of changes in transcription. Among its known mechanisms, 4-PBA can influence both transcription and translation. 4-PBA led to reduced transcription of *ABCA1* in fibroblasts, leading to decreased ABCA1 protein levels.^54^ It is possible that ABCA4 is also affected directly at the level of *ABCA4* transcription. In addition, other mechanisms could contribute to the reduction in ABCA4 protein levels following 4-PBA treatment. For example, 4-PBA has been reported to act as a weak inhibitor of mRNA translation in vitro,^55^ which might also play a role in reducing protein expression.

Overall, this study showed that these drugs are unlikely to be translated to the clinic because of both a lack of positive effects on retinal organoids and a negative effect on bystander proteins/genes. The doses used here were chosen based on to the results produced by nanoluciferase experiments in HEK cells^26^; however, different concentrations of these compounds could be effective in the rescue of ABCA4 missense variant traffic in photoreceptors, but we think this is unlikely. It is also possible that the effect of delivering the drugs directly to the retinal organoid photoreceptors through the tissue culture media would be altered by systemic delivery via the circulation or by intra-ocular routes in a tissue. Nevertheless, these data show that retinal organoids can be used to model ABCA4 disease caused by missense variants and that they are excellent tools to test small molecules as part of their translational pipeline for potential therapies.

## Supplementary Material

Supplement 1
