# Supplementary material for: Investigation of ABCA4 Missense Variants and Potential Small Molecule Rescue in Retinal Organoids
Source: Invest Ophthalmol Vis Sci. 2025 Jul 22;66(9):58. doi: 10.1167/iovs.66.9.58 (PMC12302050; doi:10.1167/iovs.66.9.58)
Supplement: Supplement 1 [file iovs-66-9-58_s001.pdf]

Supplementary materials

Investigation of ABCA4 Missense Variants and Potential Small Molecule Rescue in Retinal Organoids

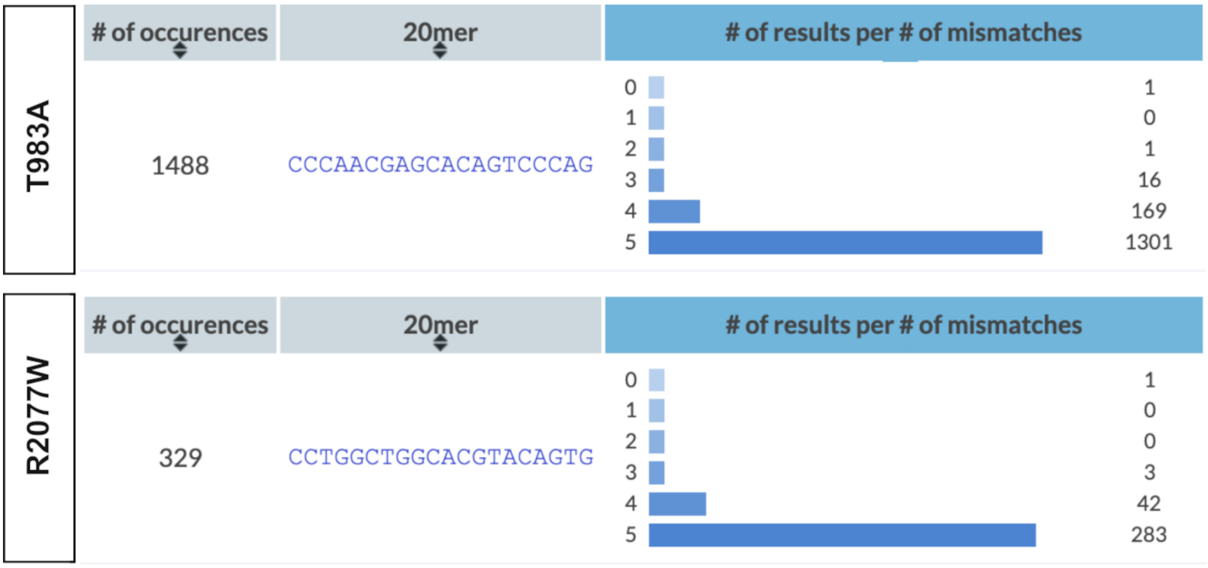

Figure S1. Off-target analysis. The total number of occurrences in the human genome (on the left), and the numbers of results per number of mismatches (on the right). 0 mismatches are found only once, corresponding to the *ABCA4* genomic region and only a number of mismatches ≥ of 2 were identified for both gRNAs.

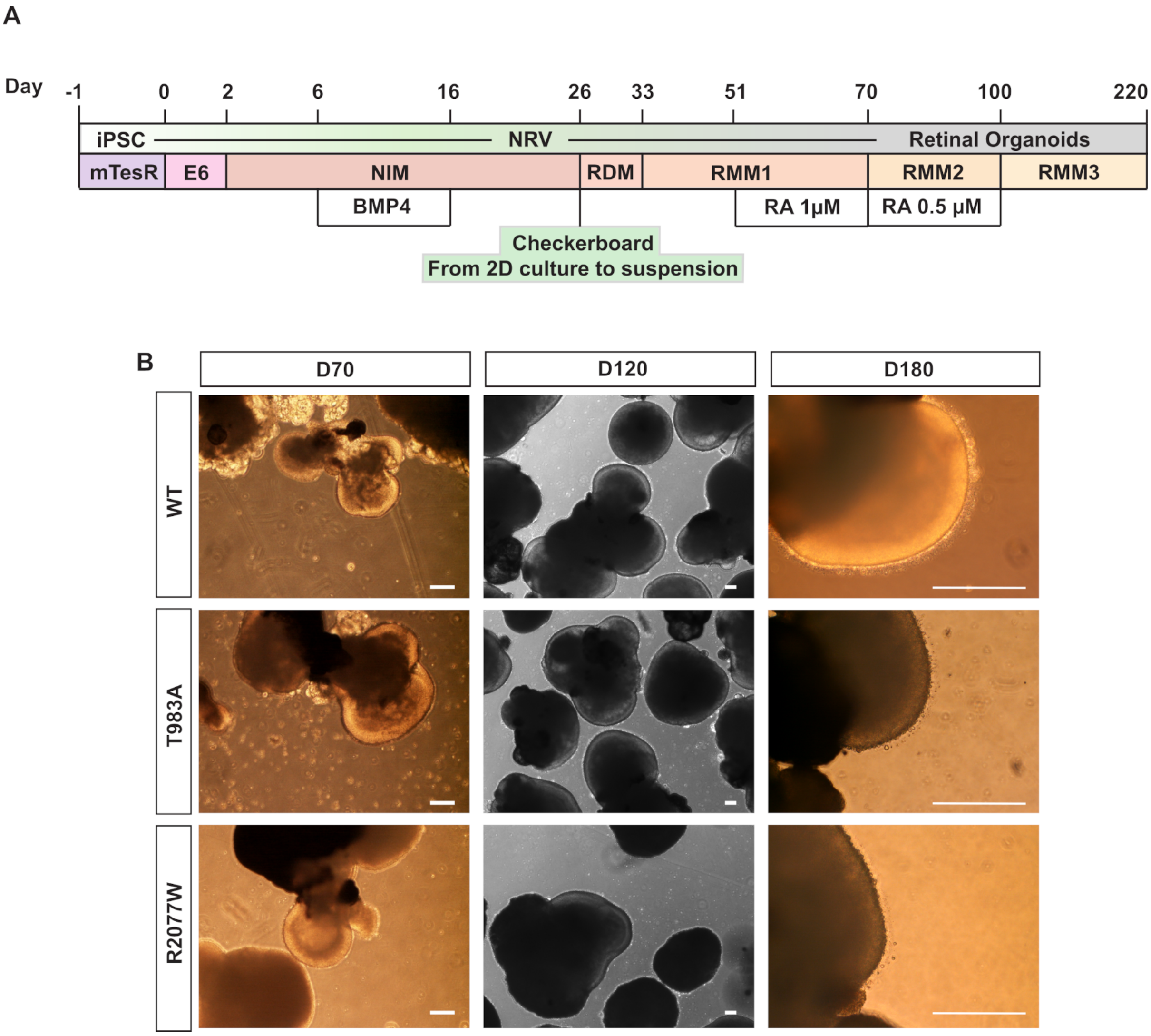

Figure S2. Generation of 3D retinal organoids.

A) Schematic timeline and media changes of a retinal organoid differentiation from iPSCs. **B)** Phase contrast images representative of 3D organoid differentiation showing morphology at indicated time points. Scale bars = 100 µm.

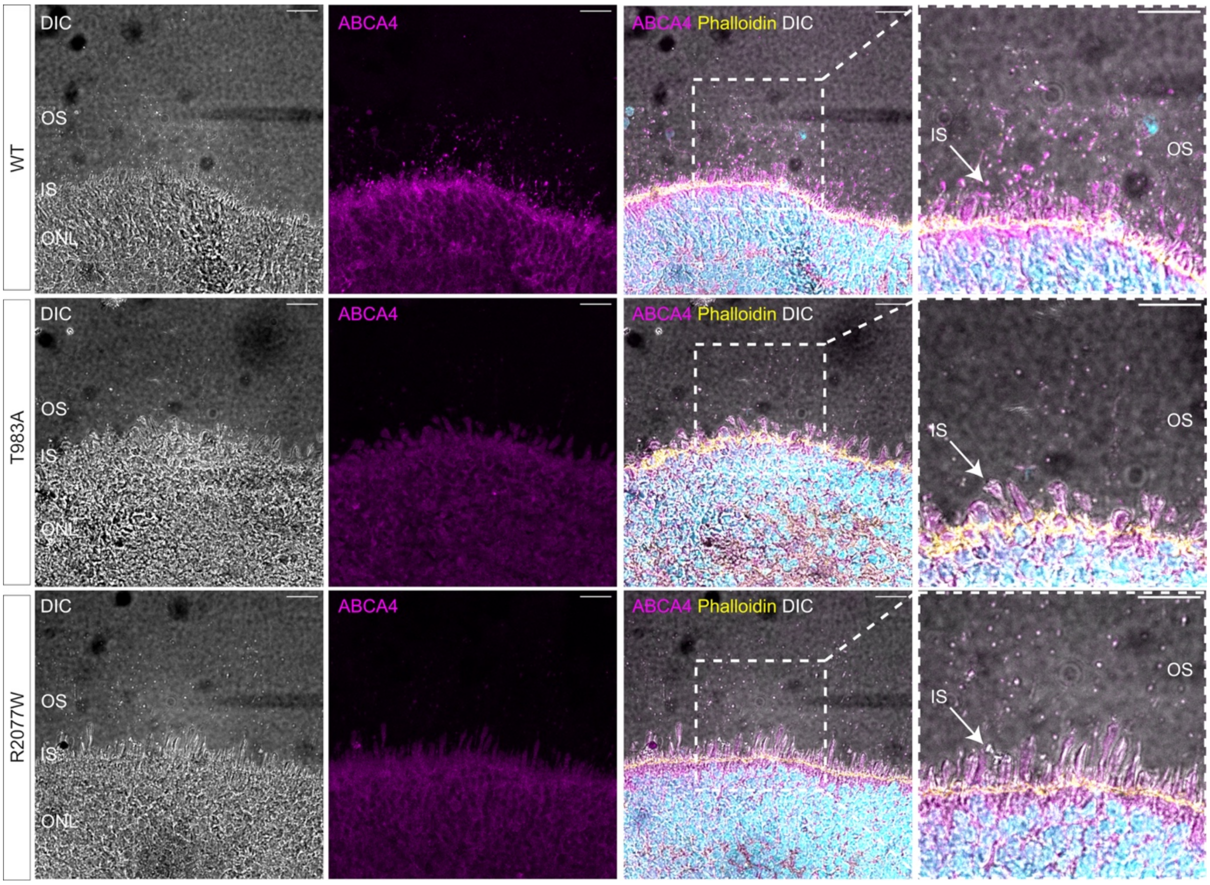

Figure S3. T983A and R2077W retinal organoids present reduced ABCA4 protein expression and reduced traffic to the OS. Confocal image of sections confirming the presence of OS using differential interreference contrast microscopy (DIC) in all the retinal organoids lines. ABCA4 3F4 C-terminus antibody clearly highlights the presence of ABCA4 protein in the IS and OS of WT retinal organoids and its absence in T983A and R2077W retinal organoids. Scale bar = 50 µm.

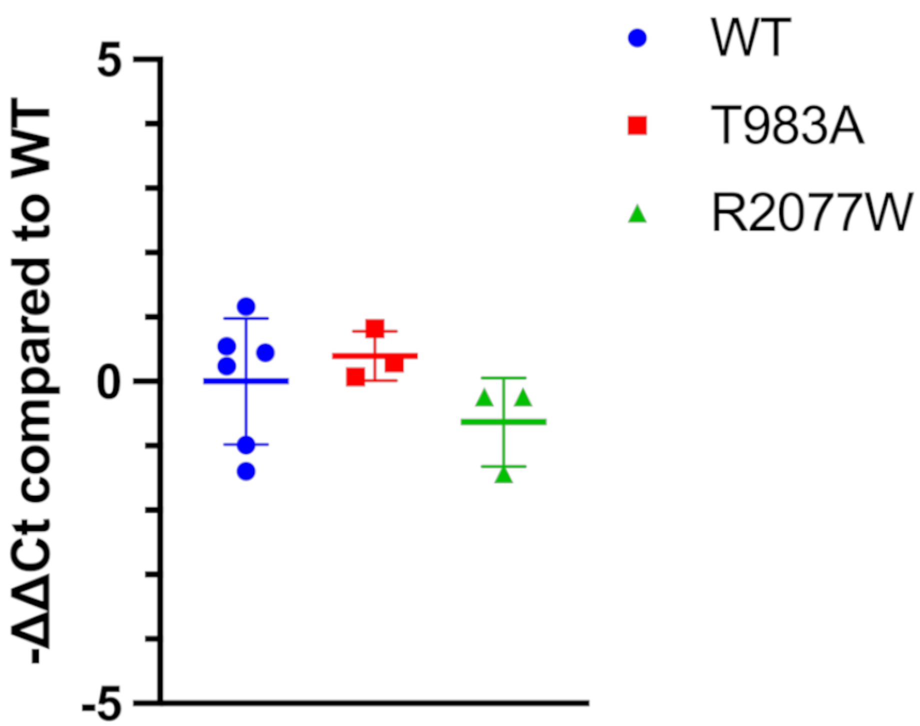

Figure S4. ***ABCA4*** **variants transcript levels are unaltered compared to WT.** *ABCA4* expression was first normalized to the geometric mean of two reference genes, GAPDH and ACTIN, before normalization to isogenic WT cell lines. Error bars are SD. One way ANOVA followed by Dunnett's post hoc test was performed only against WT ABCA4 sample.

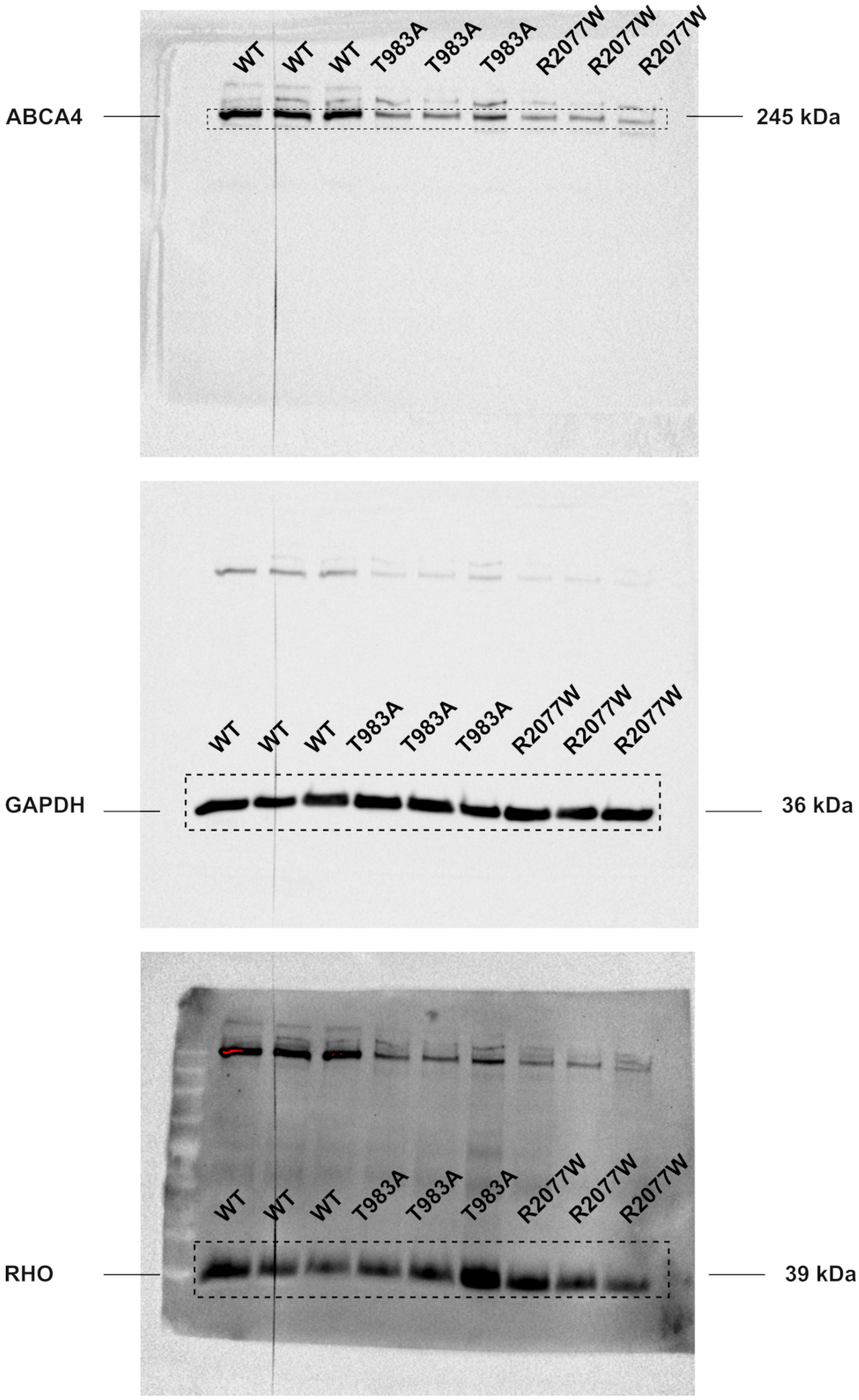

Figure S5. ABCA4 protein variants levels are reduced compared to WT. Original uncropped blots used for quantification of **Figure 3**. Box indicating the cropped area used in the main figure. n = 3. The vertical line observed is an artifact introduced by a flaw in the imaging camera we routinely used for image capture and analysis.

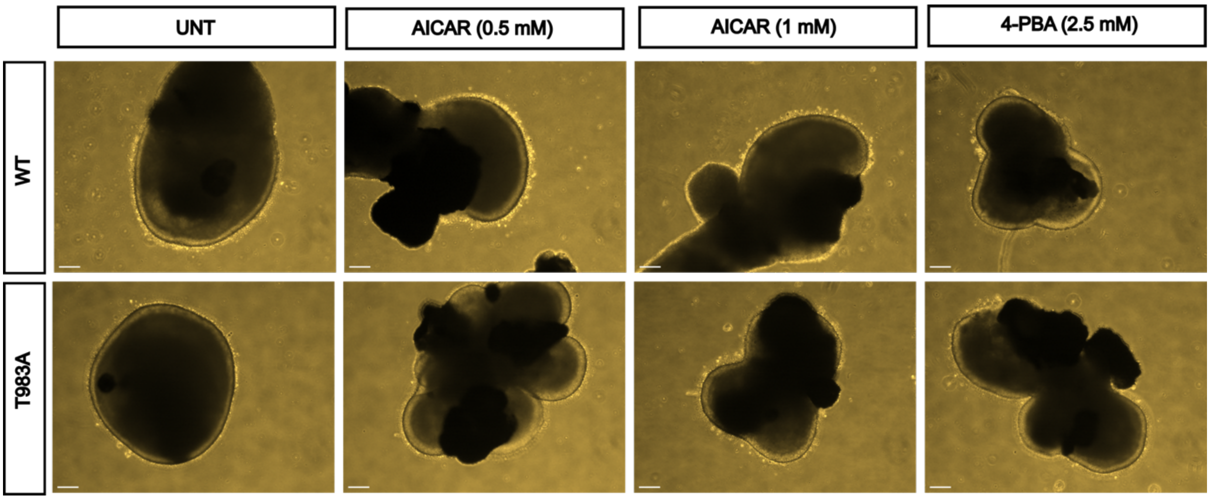

Figure S6. Treated and untreated WT and T983A retinal organoids morphology

Brightfield image of the retinal organoids pre- and post- treatment. No major morphological changes were observed. n = 3 from different differentiations. Scale bar = 50 μm

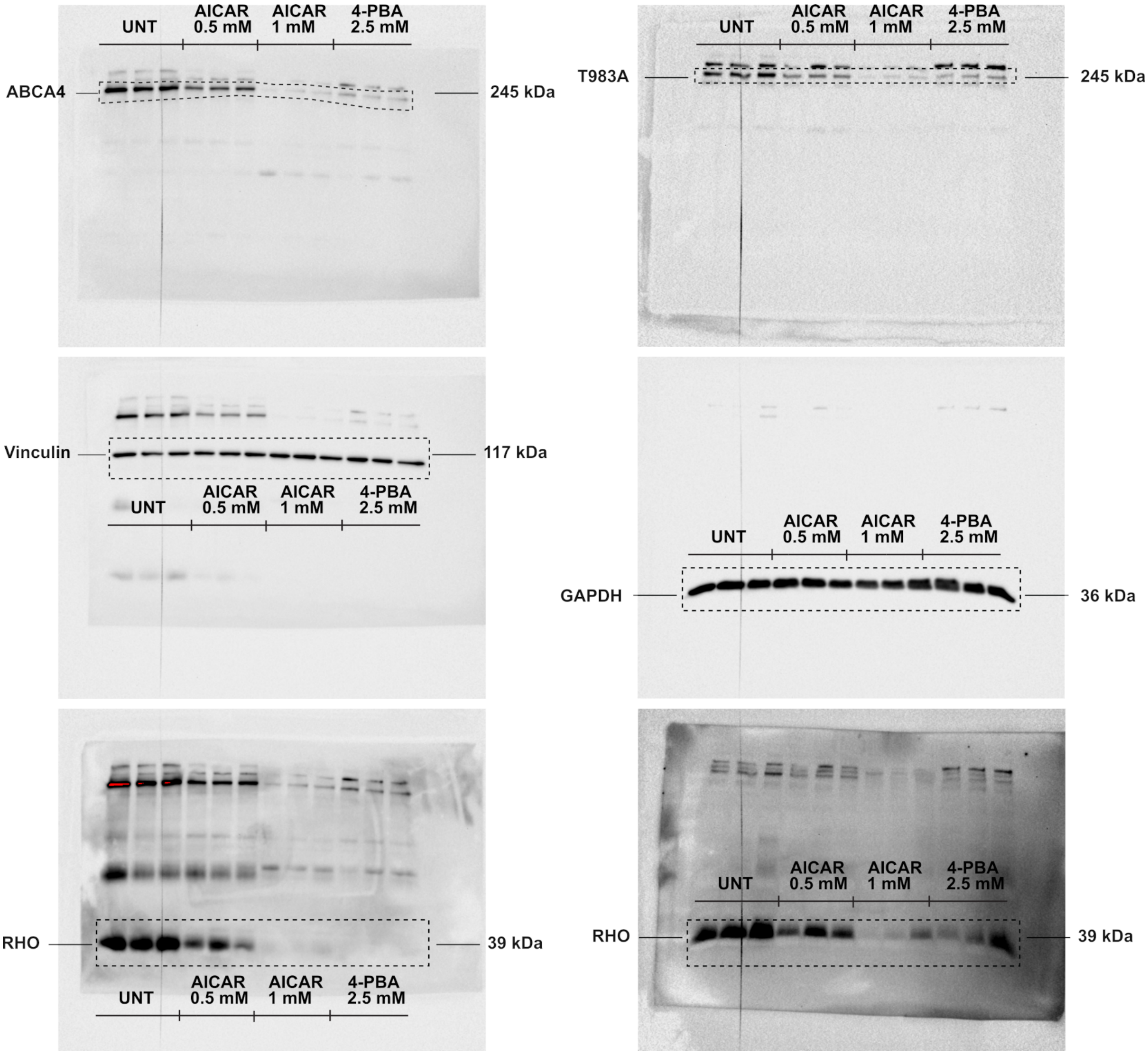

Figure S7. AICAR and 4-PBA treatments reduce ABCA4 protein levels Original uncropped blots used for quantification of **Figure 4**. Box indicating the cropped area used in the main figure. n = 3. The vertical line observed is an artifact introduced by a flaw in the imaging camera we routinely use for image capture and analysis.

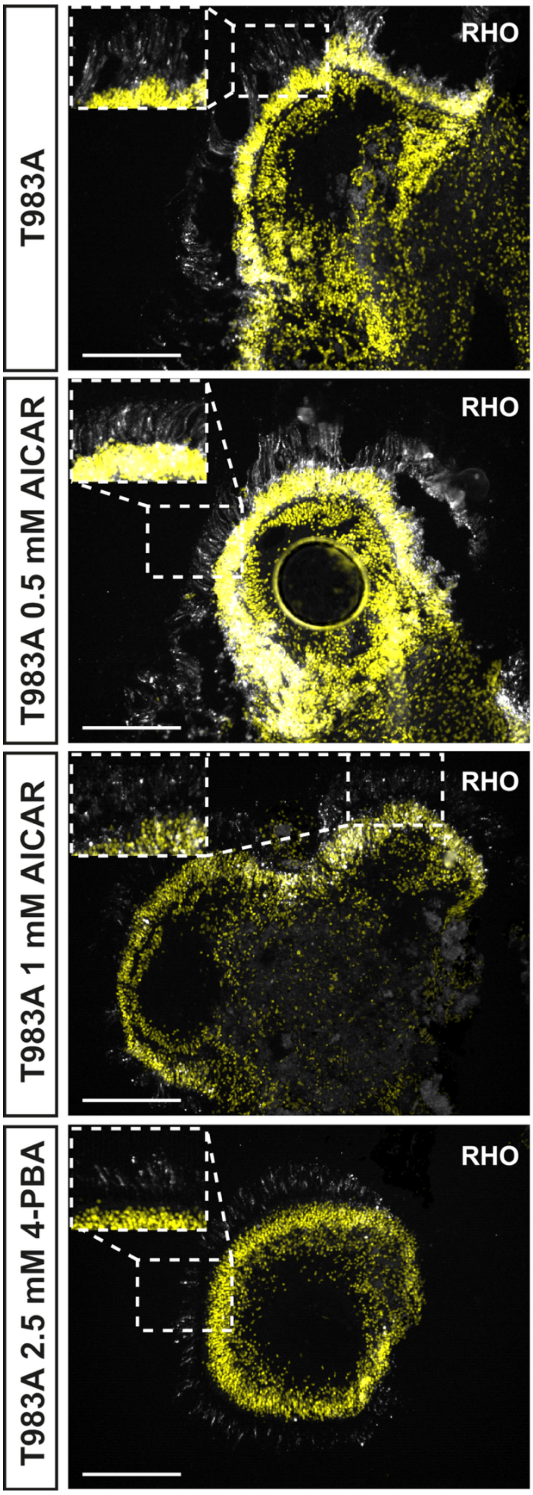

Figure S8. OS morphology of treated ABCA4 T983A retinal organoids.

Retinal organoids structure showing the laminated neuroretina-like structure with prominent outer nuclear later is visible by DAPI staining (yellow) and RHO-positive hair-like structures protruding from the laminated area, indicating a well-preserved OS. Scale bar = 200 μm.

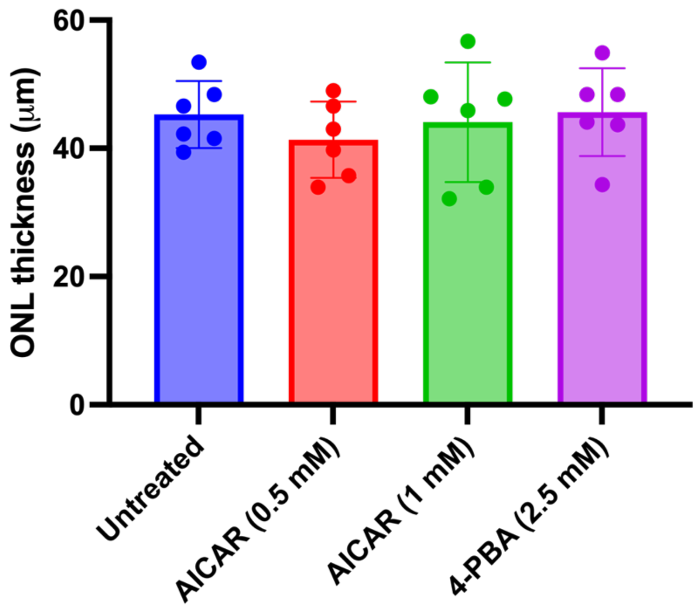

Figure S9. ONL thickness of retinal organoids. No significant change in ONL thickness was detected in treated retinal organoids compared to untreated. Measured from 6 regions from 2 different organoids, each data point represents an image.

Guide RNA	Guide oligonucleotide sequence	GC (%)	On-target score	Off-target score
**T983A**	CCCAACGAGCACAGTCCCAG	65%	73.7	41.7
**R2077W**	CCTGGCTGGCACGTACAGTG	65%	40.3	45.8

Table S1. CRISPR/Cas9 guide oligonucleotides sequences targeting *ABCA4*. gRNAs were selected for the regions of interest. On-target and off-target score was obtained via Benchling, with a higher score meaning a greater cutting efficiency and less off-targets chances. Primers are oriented 5' to 3'.

HDR	Sequence
**T983A**	CCTAAGGCATGTTGCTAAAGGCCATCTCTTCTGCCACTGACGCCTG TGTTCTGCAGGTCCATCCTGACGGGTCTGTTGCCACCAGCTTCTG GGACTGTGCTCGTTGGGGGAAGGGACATTGAAACCA
**R2077W**	TGACTGTCTACGCCGACTGCCTGGCTGGCACGTACAGTGGAGGC AACAAGTGGAAACTCTCCACAGCCATCGCACTCATTGGCTGCCCA CCGCTGGTGCTGCTGGTAACTGCGGGCTTGGGCCGCAC

Table S2. **HDR sequences targeting ABCA4**

**Gene**	**Primer**	**Product Size**
ABCA4<br>*T983A region*	Fw:<br>GGCCTGGGAGCACCATGAAAC<br>Rv:<br>CTGCTCTGTCAGAGCGAAGGC	636 base pairs
ABCA4<br>*R2077W region*	Fw: GGCGCCTGAAGCACATGCC<br>Rv:<br>GCTCCCCTGTGGAGGATGAGC	1027 base pairs

Table S3. Primers for genomic amplification

**Gene**	**Primer**		**Product Size**	**Source**
	**Forward**	**Reverse**		
ABCA4	ACCCGGAGAGAATT GCAGGA	AGACAGGCCGATGTTT TTAATGA	98	Primer Bank
ACTIN	CCAACCGCGAGAAG ATGA	CCAGAGGCGTACAGG GATA	97	-
GAPDH	CCCCACCACACTGAA TCTCC	GGTACTTTATTGATGGT ACATGACAAG	105	-

Table S4. qPCR primers
